# Combination therapy using molecular‐targeted drugs modulates tumor microenvironment and impairs tumor growth in renal cell carcinoma

**DOI:** 10.1002/cam4.1124

**Published:** 2017-08-23

**Authors:** Hiroyuki Kitano, Yasuhiko Kitadai, Jun Teishima, Ryo Yuge, Shunsuke Shinmei, Keisuke Goto, Shogo Inoue, Tetsutaro Hayashi, Kazuhiro Sentani, Wataru Yasui, Akio Matsubara

**Affiliations:** ^1^ Department of Urology Institute of Biomedical & Health Sciences Hiroshima University Hiroshima 734‐8551 Japan; ^2^ Department of Health Sciences Prefectural University of Hiroshima Hiroshima Japan; ^3^ Department of Gastroenterology & Metabolism Institute of Biomedical & Health Sciences Hiroshima University Hiroshima Japan; ^4^ Department of Molecular Pathology Institute of Biomedical & Health Sciences Hiroshima University Hiroshima Japan

**Keywords:** Combination therapy, PDGF‐R, renal cell carcinoma, stromal cell, tumor microenvironment

## Abstract

Tumor growth and metastasis are determined not by cancer cells alone but also by a variety of stromal cells, various populations of which overexpress platelet‐derived growth factor receptors (PDGF‐Rs). In addition, activation of PI3K‐AKT‐mammalian target of rapamycin (mTOR) signaling is frequently observed in many cancer types as well. mTOR comprises a serine/threonine kinase that increases the production of proteins that stimulate key cellular processes such as cell growth and proliferation, cell metabolism, and angiogenesis. In this study, we investigated the impact of molecular‐targeting agents including PDGF‐R and mTOR inhibitors on the tumor stroma of human kidney cancer and examined the efficacy of combination therapy with these agents against this disease. Treatment with sunitinib did not suppress tumor growth, but significantly decreased stromal reactivity, microvessel density, and pericyte coverage of tumor microvessels in an orthotopic mouse model. In contrast, treatment with everolimus decreased tumor growth and microvessel density but not stromal reactivity. However, sunitinib and everolimus in combination reduced both the growth rate and stromal reaction. These findings suggest that target molecule‐based inhibition of the cancer–stromal cell interaction appears promising as an effective antitumor therapy.

## Introduction

Renal cell carcinoma (RCC) accounts for 80–95% of kidney tumors exhibits poor prognosis when diagnosed at an advanced stage 1. Patients with RCC are classified according to International Metastatic RCC Database Consortium (IMDC) criteria. In particular, three or more predefined factors in the IMDC model (anemia, thrombocytosis, neutrophilia, Karnofsky performance status <80, and <1 year from diagnosis till the first targeted therapy) have been shown to represent independent predictors of poor overall survival 2. Recently, molecular‐targeted therapies for patients with metastatic RCC has become a widely recognized treatment, with approved agents including the multikinase inhibitor, sunitinib, and the mammalian target of rapamycin (mTOR) inhibitor, everolimus. Sunitinib inhibits the tyrosine kinase activity of vascular endothelial growth factor (VEGF) receptors and platelet‐derived growth factor (PDGF) receptors for use as a first‐line therapy of metastatic RCC 3, 4. In addition, treatment with sunitinib has been reported to reduce the suppressive function of myeloid‐derived suppressor cells (MDSC) and prevent T‐regulatory cells (Treg) of development 5. However, Sunitinib is not specific inhibitor for these genes. Other target molecule, c‐kit is preferentially overexpressed by GIST. Everolimus is an approved treatment from second‐line therapy after failure of tyrorine kinase inhibitor 3, 4. The mechanism of action of everolimus involves binding to mTOR complex 1 in cells to inhibit the mTOR pathway 6. According to previous reports, everolimus thereby inhibits cell proliferation as well as angiogenesis by inhibiting the expression of VEGF‐A 7, 8, 9.

Solid tumors consist of parenchyma and stroma. Recent studies in tumor biology have shown that tumor growth and metastasis are determined not only by cancer cells but also by a variety of stromal cells, and that the cancer–stromal cell interaction contributes functionally to tumor growth and metastasis 10, 11. Tumor stroma contains many different cell types including activated fibroblasts (myofibroblasts), endothelial cells, and inflammatory cells. Activated fibroblasts, termed carcinoma‐associated fibroblasts (CAFs), promote tumor growth and metastasis 12, 13, 14 and are known to be regulated via PDGFs, TGF‐*β*, and FGF‐2 15. PDGF receptors are expressed by stromal cells in addition to many human neoplastic cells of the kidney 16, prostate 17, lung 18, and colon 19. In cancers, PDGF‐R signaling is reported to increase the cell proliferation of tumor cells 20 as well as angiogenesis 21 and to regulate interstitial fluid pressure in tumor stroma 22.

Accordingly, the purpose of this study is to investigate the impact of molecular target agents on the tumor stroma of RCC and examine the efficacy of combination therapy with an mTOR inhibitor and a multiple‐tyrosine kinase inhibitor.

## Materials and Methods

### Human RCC cell lines and culture conditions

The human RCC cell line, luciferase‐transfected Caki‐1, was a gift from Dr. Peter Black (The Vancouver Prostate Center, University of British Columbia, Vancouver, Canada) and the ACHN, 786‐O, and OTU cancer cell lines were provided by Dr. Wataru Yasui (Department of Molecular Pathology, Hiroshima University, Hiroshima, Japan). The human colon cancer cell line KM12SM (which served as a negative control for PDGF‐R expression) and the human osteosarcoma cell line MG63 (which served as a positive control for PDGF‐R expression) were gifts from Dr. Yasuhiko Kitadai (Department of Gastroenterology and Metabolism, Hiroshima University, Hiroshima, Japan). The cell lines were maintained and propagated in RPMI 1640 media (Sigma‐Aldrich, St. Louis, MO) supplemented with 10% heat‐inactivated fetal bovine serum (Gibco, Gaithersburg, MD) and a penicillin–streptomycin–amphotericin B mixture at 37°C and 5% CO_2_.

### Reagents

Everolimus (RAD001) was provided by Novartis Pharma (Tokyo, Japan). Sunitinib was obtained from Pfizer (Tokyo, Japan). These compounds were diluted in sterile water for oral administration.

Primary antibodies were as follows: polyclonal rabbit anti‐PDGF‐R*β* (Santa Cruz Biotechnology, Dallas, TX); polyclonal rabbit antiphosphorylated PDGF‐R*β* (Santa Cruz Biotechnology); recombinant human PDGF subunit B homodimer (PDGF‐BB; R&D Systems, Minneapolis, MN); monoclonal rabbit antimouse mTOR antibody and monoclonal rabbit antiphosphorylated mouse mTOR antibody (Cell Signaling Technology, Danvers, MA); monoclonal rabbit antimouse S6 ribosomal protein antibody and monoclonal rabbit antiphosphorylated mouse S6 ribosomal protein antibody (Cell Signaling Technology); rat antimouse CD31 (BD Pharmingen, BD Biosciences, San Diego, CA); rabbit anti‐*α*‐smooth muscle actin (*α*‐SMA) (Abcam, Cambridge, UK); polyclonal rabbit antimouse type I collagen (Novotec, Saint Martin LaGarenne, France); monoclonal mouse antidesmin antibody from (Invitrogen; Life Technologies, Carlsbad, CA); and Ki‐67 equivalent antibody (Novocastra, Leica Microsystem, Newcastle upon Tyne, UK). Fluorescent secondary antibodies were Alexa Fluor 488‐conjugated goat antirabbit IgG, Alexa Fluor 488‐conjugated goat antirat IgG, and Alexa Fluor 546‐conjugated goat antirabbit IgG (Invitrogen; Life Technologies, Carlsbad, CA).

### Cell proliferation assay

In vitro growth was assessed using a colorimetric assay with 3‐(4,5‐dimethylthiazol‐2‐yl)2,5‐diphenyltetrazolium bromide (MTT). Caki‐1 cells were seeded in a 96‐well plate at a density of 1000 cells/well and incubated overnight in 100 *μ*L RPMI 1640 containing 10% FBS at 37°C in a humidified atmosphere of 5% CO_2_ and 95% air. After incubation for 24 h, the medium was replaced with RPMI 1640 containing 10% FBS and specified doses of everolimus (0.1, 0.5, 1 nmol/L) and sunitinib (0.1, 0.5, 1 *μ*mol/L). MTT solution was added after the plates had been incubated for 3 days, with an additional 3 h incubation at 37°C. The plates were then analyzed using an ELISA plate reader (Bio‐Rad, Hercules, CA) at 570 nm with the reference wavelength of 630 nm. Each experiment was repeated three times and the data were recorded as the mean and SD.

### Western blot analysis

To examine whether everolimus or sunitinib affects the mTOR pathway, Caki‐1 cells were cultured in RPMI 1640 containing 10% FBS for 24 h and incubated for 2 days in medium with or without everolimus (20 nmol/L) or sunitinib (20 *μ*mol/L).

To examine whether these drugs affect the PDGF pathway, Caki‐1 cells were cultured in serum‐free culture medium for 1 h and then stimulated with 10 ng/mL PDGF‐BB for 15 min. Cells were harvested by scraping in Tris‐glycine sodium dodecyl sulfate sample buffer (Invitrogen). The proteins of harvested cells were separated by sodium dodecyl sulfate‐polyacrylamide gel electrophoresis and electrotransferred onto nitrocellulose filters. The immune complexes generated following hybridization with anti‐PDGF‐R*β* (1:50) or anti‐pPDGF‐R*β* (1:200) primary antibodies were visualized by enhanced chemiluminescence with an ECL Western Blot Detection System (Amersham Biosciences, Piscataway, NJ). *β*‐Actin (Sigma) was also stained as a loading control.

### Animals and orthotopic implantation of tumor cells

Female athymic nude BALB/c mice were obtained from Charles River Japan (Tokyo, Japan). The mice were maintained under specific pathogen‐free conditions and used at 5–6 weeks of age. The study was conducted with permission from the Committee on Animal Experimentation at our University. Mice were anesthetized with somnopentyl, and their backs were prepared for sterile surgery. A small back incision was made, and the left kidney was exteriorized. To produce renal tumors, Caki‐1 cells (1 × 106) in 30 *μ*L of Hanks’ balanced salt solution were injected into the capsule of the left kidney with a 30‐gauge needle under observation with a magnifying glass.

### Treatment of established human kidney cancer tumors growing in nude mice

At 14 days after cell implantation, 10 mice were randomly assigned to each of four treatment groups: (1) daily administration of water by oral gavage (control), (2) daily oral gavage of 1 mg/kg everolimus, (3) daily oral gavage of 10 mg/kg sunitinib, or (4) daily oral gavage of 1 mg/kg everolimus + 10 mg/kg sunitinib. Combination studies with everolimus and sunitinib have not frequently been performed because of drug–drug interactions that would affect the pharmacokinetic profiles and tolerability of the agents in vivo. In this study, we intended to observe the stromal reaction of RCC in detail. Accordingly, the everolimus or sunitinib dosage was reduced compared to that in single‐agent therapy, with the everolimus and sunitinib dosage reduced to 20% and 25% of the quantity required for maximal durability, respectively 23.

### Evaluation of kidney tumors in nude mice using the Perkin Elmer In Vivo Imaging System^®^ (IVIS Caliper)

d‐luciferin (150 mg/kg, Perkin Elmer, Waltham, MA) was injected intraperitoneally into mice previously implanted with Caki‐1 cells. Bioluminescence was detected and measured using IVIS. Living Image Software (Xenogen, PerkinElmer, Inc., Waltham, MA) was used to quantify the photons per second emitted by the cells. Bioluminescence was measured and quantified at 15 min after injection of d‐luciferin using a subject height of 1.5 cm, medium binning, and an exposure time of 0.5 s/min prior to and following drug administration after 14 and 28 days.

### Necropsy and histological studies

Mice bearing orthotopic tumors were euthanized using diethyl ether. Body weights were recorded. After necropsy, tumors were excised and weighted.

For immunohistochemistry, one part of the tumor tissue was fixed in formalin and embedded in paraffin. The other part was embedded in Tissue‐Tek OCT compound (Sakura Finetek, Torrance, CA), rapidly frozen in liquid nitrogen, and stored at −80°C.

$$\text{Tumor volume was calculated as V}=1/2(\text{length}\times {\text{width}}^{2}).$$

### Double immunofluorescence staining for PDGF‐R*β* and *α*‐SMA (CAFs) or desmin (pericytes)

Fresh‐frozen specimens of Caki‐1 human renal cell carcinoma tissue obtained from nude mice were cut into 8‐*μ*m sections and mounted on positively charged slides. In preparation for the assays, sections were fixed in ice‐cold acetone for 20 min and washing and blocking were performed as previously described 22. The slides were incubated overnight at 4°C with primary antibodies against *α*‐SMA (1:50) and desmin (1:400). Slides were then rinsed three times with phosphate buffered saline (PBS) and incubated for 10 min in protein blocking solution. For desmin staining, slides were incubated overnight at 4°C with Fab fragment goat antimouse IgG (Jackson ImmunoResearch Laboratories, West Grove, PA) to block endogenous immunoglobulins. This was followed by a short incubation with protein blocking solution and then by incubation with primary antibody. Slides were then incubated for 1 h at room temperature with Alexa 488‐conjugated secondary antibody followed by overnight incubation at 4°C with an antibody against PDGF‐R*β*. The sections were rinsed three times with PBS and incubated for 10 min in protein blocking solution. Subsequently, slides were incubated for 1 h at room temperature with Alexa 546‐conjugated goat antirabbit IgG secondary antibody. The samples were then rinsed three times in PBS and nuclear counterstained with DAPI for 10 min. Samples were again rinsed three times with PBS and mounting medium was placed on each sample, which was protected with a glass coverslip. The fluorescent signal of the secondary antibody was captured by confocal laser scanning microscopy (Carl Zeiss Microscopy, Thornwood, NY; Jena, Germany). CAFs or pericytes were identified by green fluorescence, whereas PDGF‐R*β* was identified by red fluorescence.

### Double immunofluorescence staining for CD31 (vascular endothelial cells) and desmin (pericytes)

To identify endothelial cells, slides were incubated overnight at 4°C with an antibody against CD31. This was followed by incubation with Alexa 546‐conjugated goat antirat IgG secondary antibody, and the slides were again blocked in a blocking solution as described above and incubated with antibody against desmin. After further washing and further blocking with blocking solution, the slides were incubated with Alexa 488‐conjugated goat antirabbit IgG secondary antibody. Endothelial cells were identified by red fluorescence. The coverage of pericytes on endothelial cells was determined by counting CD31‐positive cells in direct contact with desmin‐positive cells in five randomly selected microscopic field (at 100× magnification).

### Immunohistochemical staining and immunofluorescence staining

Formalin‐fixed, paraffin‐embedded tissues cut into serial 4 *μ*m sections were used for immunohistochemistry. The procedures for immunohistochemical detection of phosphorylated PDGF‐R, phosphorylated S6 ribosomal protein (p‐S6), Ki‐67, *α*‐SMA, and type I collagen were as described previously. The Ki‐67 labeling index (Ki‐67 LI) was determined by light microscopy at the site of the greatest number of Ki‐67^+^ cells. Cells were counted in five fields at ×100 magnification and the number of Ki‐67^+^ cells among approximately 300 tumor cells was counted and expressed as a percentage.

The transplanted tumor tissues were prepared into 8 *μ*m frozen sections and then were subjected to immunofluorescence analyses using a primary antibody for CD31. Vascular endothelial cells were identified by red fluorescence.

### Quantification of microvessels, CAFs, and type‐1 collagen areas

To evaluate the angiogenic activity of the tumors, microvessel (CD31^+^) areas were quantified. We captured 10 random fields at ×200 magnification for each tumor and the vessels including the lumen were counted manually. CAFs were counted at *α*SMA^+^ cells and extracellular matrix was also determined from the respective areas of type I collagen^+^ staining from 10 optical fields (×100 magnification) of different sections. The areas were calculated with the use of ImageJ software version 1.47v (National Institutes of Health, Bethesda, MD).

### Confocal microscopy

Confocal fluorescence images were collected using 10×, 20×, and 40× objective lenses on a Zeiss LSM laser scanning microscopy system (Carl Zeiss) equipped with a motorized Axioplan microscope, argon laser (458/477/488/514 nm, 30 mW), HeNe laser (543 nm, 1 mW), HeNe laser (633 nm, 5 mW), LSM 510 control and image acquisition software, and appropriate filters (Chroma Technology Corp., Brattleboro, VT). Confocal images were exported into Adobe Photoshop and montages were prepared for publication photos.

### Statistical analysis

Between‐group differences in murine body weight, tumor weight, luciferase activity, and *α*‐SMA^+^ cells, areas of type I collagen^+^, CD31^+^ cells, were analyzed using the Mann–Whitney/Kruskal–Wallis test. Differences in the percentages of Ki‐67^+^ cells were analyzed using the Mann–Whitney test. *P *<* *0.05 was considered statistically significant. Data are expressed as the mean ± SEM.

## Results

### Effects of everolimus or sunitinib on the phosphorylation of targeted proteins in human kidney cancer cell lines

**Figure 1:**
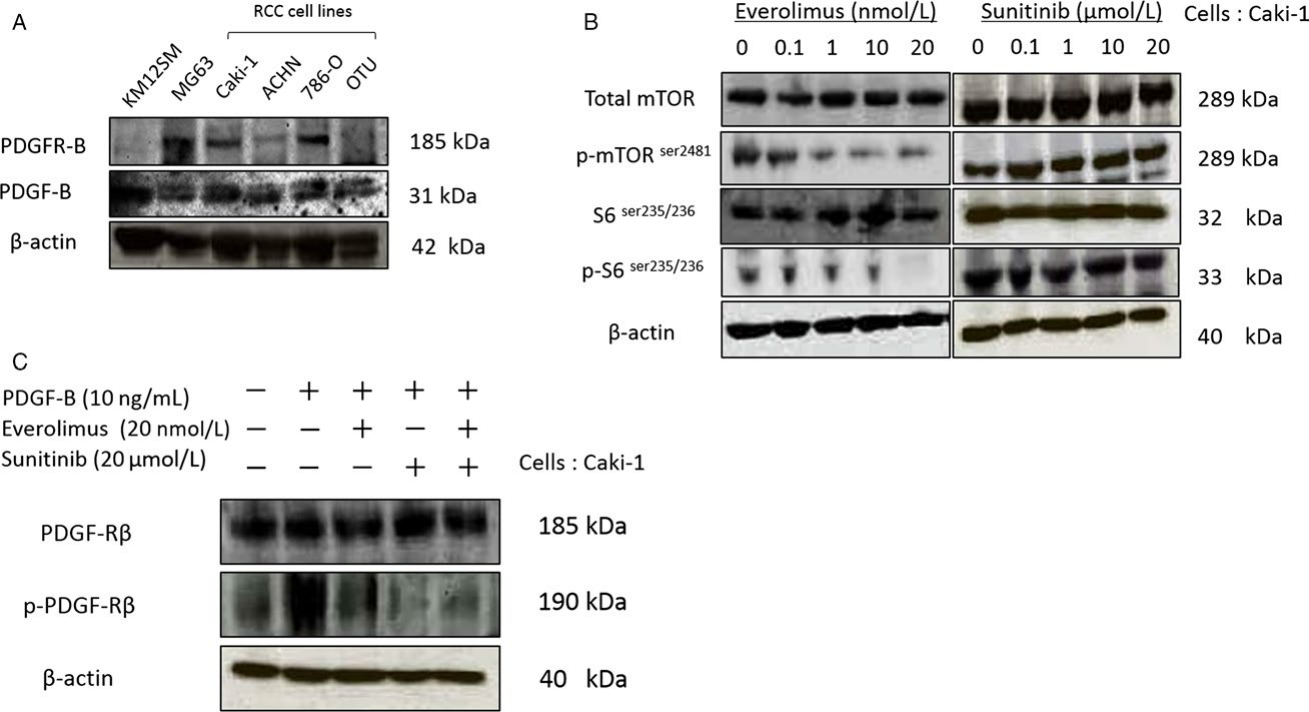
Effect of everolimus and/or sunitinib on mTOR and platelet‐derived growth factor (PDGF) signaling. (A) Western blot analyses for expression of PDGF‐B in RCC cell lines. (B) Assessment of phosphorylated mTOR and p‐S6 levels following treatment of Caki‐1 cells with everolimus or sunitinib for 48 h. (C) Assessment of phosphorylated PDGFR following treatment of PDGF‐B‐stimulated Caki‐1 cells with everolimus or sunitinib.

We examined the expression of PDGF‐B and PDGF‐R*β* in four human kidney cancer cell lines (Caki‐1, ACHN, 786‐O, and OUT). The human colon cancer cell line KM12SM and human osteosarcoma cell line MG63 were also used as negative and positive controls for PDGF‐R*β*, respectively. All kidney cancer cell lines expressed both PDGF‐B proteins in vitro, whereas KM12SM and OTU did not express PDGF‐R*β* (Fig. 1A).

We next obtained Caki‐1 cells transfected with luciferase, which allowed us to utilize in vivo tumorography and were used for the following in vitro and in vivo experiments. To examine the effect of everolimus on mTOR signaling in vitro, western blot analysis was performed on Caki‐1 cell lysate samples. S6 and mTOR phosphorylation were inhibited in a dose‐dependent manner after 48 h of everolimus (20 nmol/L) treatment. In contrast, phosphorylation of S6 and mTOR was not affected by treatment with sunitinib (20 *μ*mol/L) (Fig. 1B). We next examined the effect of sunitinib on phosphorylation of PDGFR*β* in vitro. Following everolimus treatment, no effect was observed on the PDGF pathway in Caki‐1 cells; however, sunitinib inhibited the phosphorylation of PDGFR*β* stimulated by PDGF‐B, although it did not influence the effect of everolimus on the mTOR pathway (Fig. 1C).

### Effects of everolimus and sunitinib on the cell proliferation of Caki‐1 cells in cell culture

**Figure 2:**
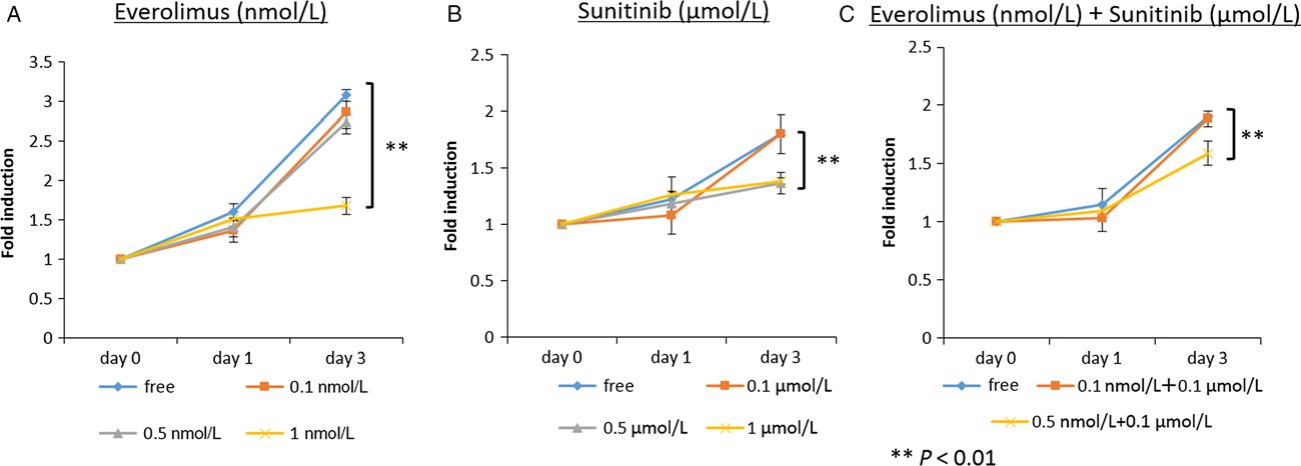
Growth of Caki‐1 cells following everolimus (A), sunitinib (B), or everolimus + sunitinib treatment (C). Cell growth was inhibited with 1 nmol/L everolimus or 0.5 *μ*mol/L sunitinib as well as by 0.5 nmol/L everolimus +0.1 *μ*mol/L sunitinib.

To assess the effects of everolimus and sunitinib on the growth of Caki‐1 cells in vitro, a cell proliferation assay was conducted. Caki‐1 cells were treated with everolimus (0–1 nmol/L) or sunitinib (0–1 *μ*mol/L) for 72 h. The two drugs exhibited a dose‐dependent growth inhibition of Caki‐1 cells, as shown in Fig. 2A and B. Combination therapy with everolimus and sunitinib also exhibited a dose‐dependent growth inhibition of Caki‐1 cells. Single‐agent treatments using everolimus (0.5 nmol/L) or sunitinib (0.1 *μ*mol/L) did not inhibit the cell proliferation; however, the combination therapy (everolimus; 0.5 nmol/L +  sunitinib; 0.1 *μ*mol/L) was effective for cell growth inhibition (Fig. 2C).

### Effect of everolimus and sunitinib on Caki‐1 tumor growth in the kidneys of nude mice

**Figure 3:**
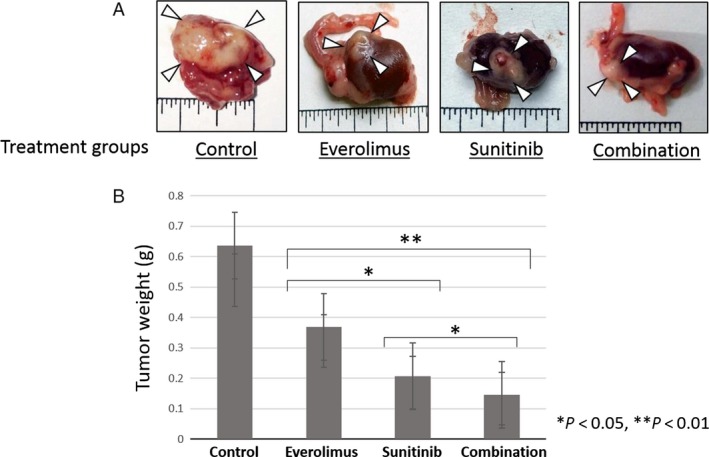
Effects of everolimus and/or sunitinib on Caki‐1 orthotopically implanted kidney tumors. (A) Macrophotograph of orthotopically implanted kidney tumors. (B) Tumor weights between the various treatment groups.

We measured the weights of the host body and of orthotopically implanted tumors to evaluate the effects of the molecular target drugs (Table 1). The tumor incidence was 100% in all four treatment groups. Body weights of the mice were measured prior to administration of the drugs and at 14 and 28 days after administration. Body weights were significantly reduced in the control group as compared to the groups treated with drugs. Tumor weights were measured at necroscopy. As compared to the control group, tumor weights were significantly reduced in the treatment groups (Fig. 3B). The tumor weight of the mice treated with sunitinib was much lower than that of the mice treated with everolimus and tumor weight was reduced in the mice treated with both everolimus and sunitinib (Table 2).

**Table 1:** Human RCC cell lines and Reagents

Human cell lines	
The human colon cancer cell line	KM12SM
The human osteosarcoma cell line	MG63
The human RCC cell line	Caki‐1
	ACHN
	786‐O
	OTU

Reagents				
		WB	IHC	IF
Primary antibody	Polyclonal rabbit anti‐PDGF‐R*β*	1:50		1:50
	Antiphosphorylated PDGF‐R*β*	1:200	1:50	
	Monoclonal rabbit antimouse mTOR antibody	1:1000		
	Monoclonal rabbit antiphosphorylated mouse mTOR antibody	1:1000		
	Monoclonal rabbit antimouse S6 ribosomal protein antibody	1:1000		
	Monoclonal rabbit antimouse S6 ribosomal protein antibody	1:1000		
	Monoclonal rabbit antiphosphorylated mouse S6 ribosomal protein antibody	1:1000	1:100	
	Rat antimouse CD31			1:25
	Rabbit anti‐*α*‐smooth muscle actin		1:50	1:50
	Polyclonal rabbit antimouse type I collagen		1:500	
	Monoclonal mouse antidesmin antibody			1:400

**Table 2:** Results of treatment of Caki‐1 tumors growing in the nude mice

Treatment group	Body weight, g median (range)	Tumor incidence, *n*/*N*	Tumor weight, g median (range)
Control	11.7 (9.0–16.0)	10/10	0.35 (0.24–1.26)
Everolimus (1 mg/Kg)	18.5 (14.7–23.3)	8/8	0.24 (0.16–0.55)
Sunitinib (10 mg/Kg)	21.2 (19.6–23.0)	9/9	0.13 (0.08–0.28)
Everolimus (1 mg/Kg) + Sunitinib (10 mg/Kg)	20.8 (18.7–23.2)	10/10	0.10 (0.08–0.19)

**Figure 4:**
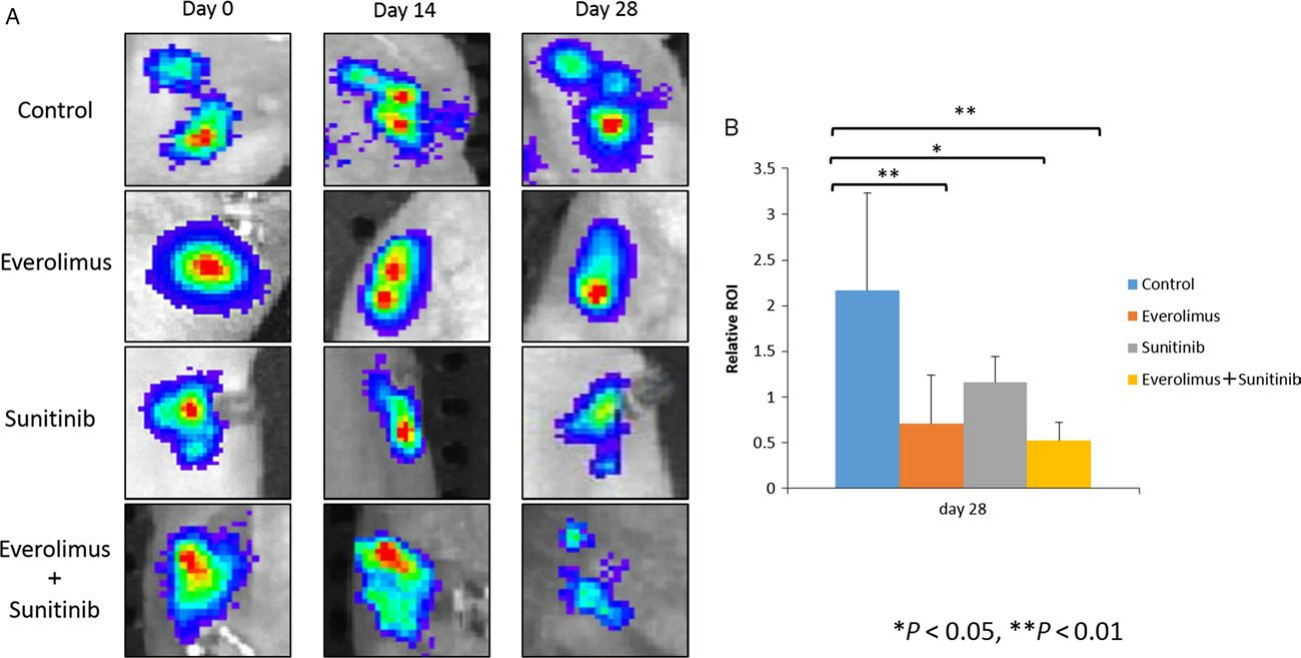
Bioluminescence images of mice from each treatment group are shown prior to treatment and at 14 or 28 days after treatment. The number of cancer cells was determined by region of interest (ROI) analysis of total photons per second.

We also monitored the luciferase activity of Caki‐1 cells using IVIS (Fig. 4A). Luciferase activities were significantly reduced in the groups treated with drugs as compared to that of the control group and the luciferase activity of the mice treated with everolimus was much lower than that of the mice treated with sunitinib (Fig. 4B).

### Effects of everolimus and sunitinib on PDGF and mTOR signaling pathways in orthotopic tumors

**Figure 5:**
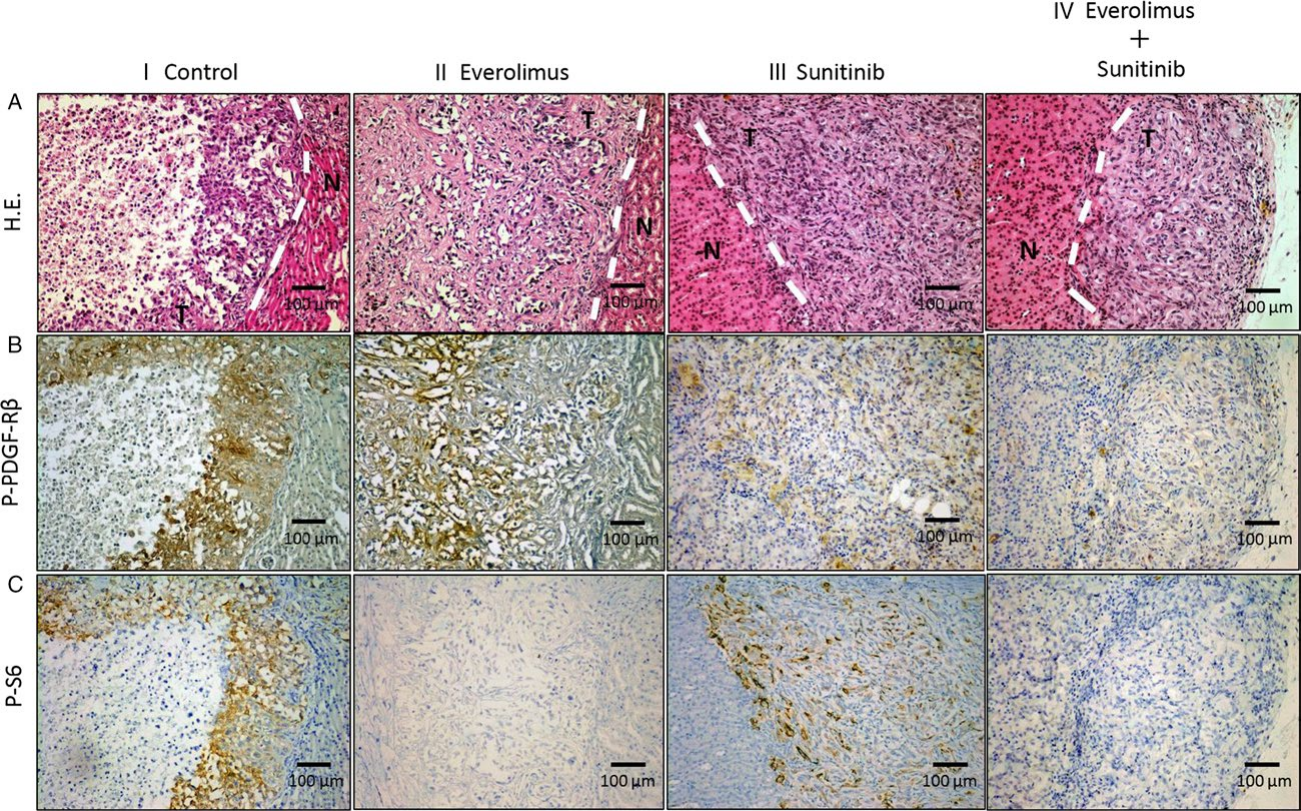
Immunohistochemistry for p‐PDGF‐R*β*, p‐S6, and Ki‐67. Mice with kidney Caki‐1 tumors were treated with (i) vehicle (control), (ii) everolimus, (iii) sunitinib, or (iv) everolimus + sunitinib. (A) Hematoxylin and eosin (H.E.) stain. (B) Phosphorylation of PDGF‐R*β* in tumor nests as measured by treatment with sunitinib or everolimus and sunitinib in combination. (C) Phosphorylation of S6 in tumor nests as measured by treatment with everolimus or everolimus and sunitinib in combination. Data are expressed as the mean ± SEM. *P <* *0.01, **P <* *0.05. Scale bars: 100 *μ*m (A–C).

To assess whether everolimus or sunitinib inhibited the phosphorylation of targeted proteins, tumor specimens were analyzed using immunohistochemistry for the expression of p‐PDGF‐R*β* and p‐S6. Phosphorylation of PDGF‐R was inhibited in orthotopic tumors of mice treated with sunitinib alone or with everolimus and sunitinib in combination (Fig. 5B). Phosphorylation of S6 ribosomal protein was markedly inhibited in the group treated with everolimus alone or with everolimus and sunitinib in combination as compared to the sunitinib group (Fig. 5C).

### Expression of PDGF‐R*β* in CAFs and pericyte localization of PDGF‐R*β* in orthotopic tumors generated using Caki‐1 cells

**Figure 6:**
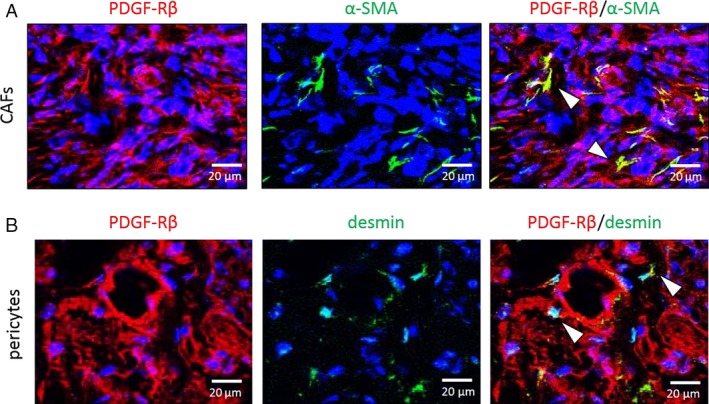
Fluorescence double‐labeled immunohistochemistry (IHC) of Caki‐1 human kidney cancer cells grown in nude mice. Representative images show IHC for *α*‐SMA (CAFs marker, Fig. 6A), desmin (pericyte marker, Fig. 6B) in green, and PDGFR‐*β* in red. Expression of PDGFR‐*β* in CAFs or pericytes is indicated by an arrow‐head. Scale bars: 20 *μ*m (A, B).

To identify whether CAFs or pericytes express PDGF‐R*β*, we performed colocalization analyses of PDGF‐R*β* and *α*‐SMA (CAFs) or desmin (pericytes). The co‐localization of green (*α*‐SMA or desmin) and red (PDGF‐R*β*) fluorescence yielded yellow fluorescence, indicating that CAFs or pericytes expressed PDGF‐R*β* (Fig. 6A and B).

### Determination of the Ki‐67 LI

**Figure 7:**
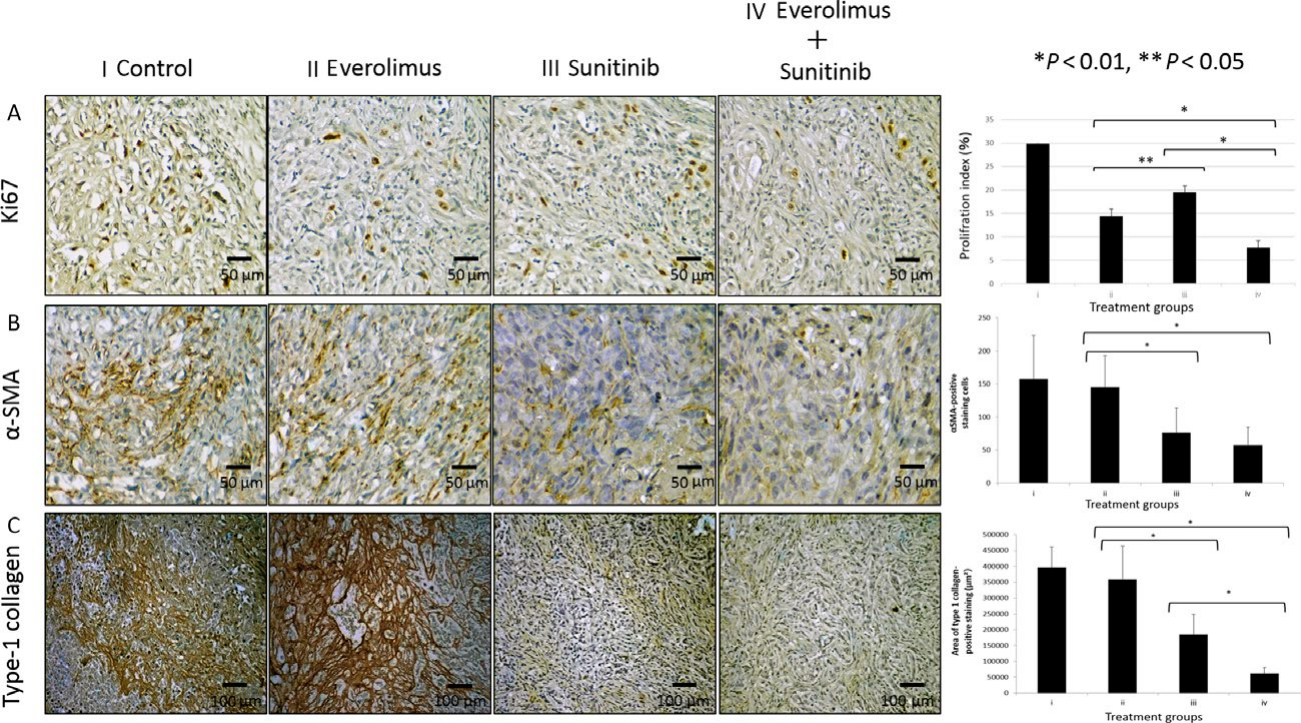
Immunohistochemistry for Ki‐67, CAF, and Type‐1 collagen. Mice with kidney Caki‐1 tumors were treated with (i) vehicle (control), (ii) everolimus, (iii) sunitinib, or (iv) everolimus + sunitinib. (A) Analysis of cell proliferation (Ki‐67), (B) CAFs (*α*‐SMA), or (C) extracellular matrix (Type‐1 collagen). Data are expressed as the mean ± SEM. *P <* *0.01, **P <* *0.05. Scale bars: 50 *μ*m (A, B); 100 *μ*m (C).

Next, we examined effect of drug therapy on tumor cell proliferation (Fig. 7A). The Ki‐67 LI was lower in the treated groups than in the control group and decreased under treatment with everolimus or sunitinib alone as well as under treatment with everolimus and sunitinib in combination. However, the Ki67 LI was significantly decreased in the everolimus alone or combination groups as compared to the sunitinib group.

### Inhibition of Caki‐1 tumor stromal reactivity by sunitinib but not everolimus

Caki‐1 cells orthotopically implanted into the capsule of the left kidney grew invasively regardless of everolimus treatment, whereas with sunitinib treatment the tumors grew expansively (Fig. 5A). The stromal reaction in tumors was evaluated as the counts of CAFs and the areas of type‐I collagen positivity. CAFs were significantly reduced in the sunitinib group compared with the control and everolimus groups (*P *<* *0.01). Furthermore, in the combination therapy group, CAFs were significantly reduced and to a greater degree than in the everolimus group (*P *<* *0.01, Fig. 7B). The area of type‐I collagen was also significantly reduced in the sunitinib group compared with that in the control and everolimus groups (*P *<* *0.01). Additionally, in the combination therapy group, the areas were significantly reduced to a greater degree than those in the monotherapy group (*P *<* *0.01, Fig. 7C).

### Effect of the molecular target drugs on the tumor vascular phenotype

**Figure 8:**
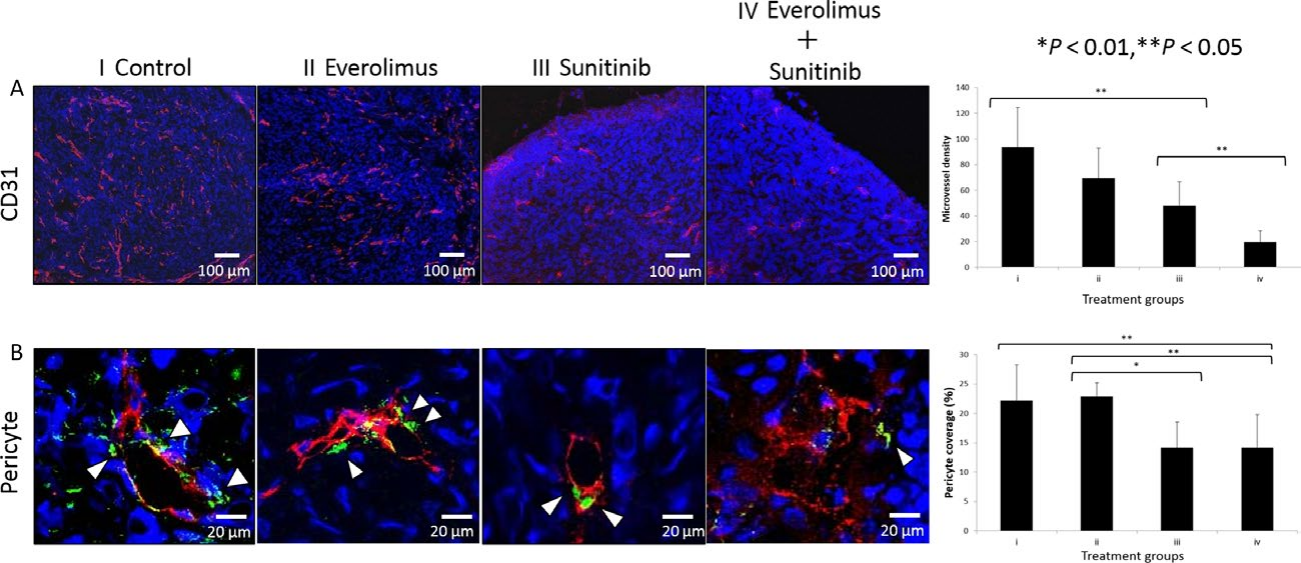
Effects of everolimus and/or sunitinib on Caki‐1 orthotopic tumors. (A) Angiogenesis (CD31). (B) Vascular endothelial cells were identified by red fluorescence and pericytes by green fluorescence. Data are expressed as the mean ± SEM. *P <* *0.01; **P <* *0.05. Scale bars: 100 *μ*m (A), 20 *μ*m (B).

Microvessel density decreased with everolimus and sunitinib compared with that in the control group. However, the difference between the control and everolimus groups was not significant. In the combination therapy group, microvessel density significantly decreased to a greater degree than that in the monotherapy groups (*P *<* *0.05) (Fig. 8A).

The effects of drug treatments on pericyte coverage of endothelial cells were evaluated by double immunofluorescence staining with anti‐CD31 and antidesmin antibodies. We noted morphologic differences between pericytes in the control versus everolimus groups and in the sunitinib versus combination groups. In the control or everolimus group, pericytes were interspersed with tumor‐associated endothelial cells; however, in the sunitinib or combination group this phenomenon was absent. The number of pericytes covering endothelial cells decreased in the sunitinib or combination group compared with that in the control or everolimus group (Fig. 8B).

## Discussion

Here, we showed that sunitinib (a multityrosine kinase inhibitor) mainly reduces the stromal reaction in RCC and that everolimus decreases cell proliferation to a greater degree than sunitinib. However, in combination, these molecular target drugs significantly inhibited tumor growth, stromal reaction, and angiogenesis at the site of orthotopic implantation (kidney).

Sunitinib inhibits the tyrosine kinase activity of VEGF receptors, PDGF receptors, c‐kit, and RET, thereby inhibiting angiogenesis and lymphangiogenesis by blockade of VEGFR pathway, and tumor cell proliferation of GIST by blockage of c‐kit 16, 24, 25. Furthermore, it has been reported that treatment with sunitinib decreased the number of MDSC and Treg in advanced tumor‐bearing animals 5. In this study, we found PDGF receptors in RCC cell lines. Therefore, sunitinib may inhibit cell proliferation of RCC in addition to stromal inhibition. In colorectal cancer, CAFs and pericytes express PDGF‐R*β* 26, 27, 28; in turn, PDGFs reportedly produced by cancer cells activate malignant interstitial tissues and contribute to tumor growth, invasion, and metastasis 29. Furthermore, it has been reported that in breast and prostate cancers, the expression of PDGF‐Rs is associated with prognosis. Specifically, high PDGF‐R*β* expression was associated with a significantly shorter recurrence‐free survival and breast cancer‐specific survival. In addition, high tumor and nonmalignant stromal PDGF‐R*β* immunostaining was associated with an increased relative risk for prostate cancer‐specific death 30, 31. However, in RCC, many aspects of interstitial tissues have not been elucidated other than the abundant angiogenesis that occurs during the carcinogenic process owing to mutation and deletion of the von Hippel‐Lindau gene. Thus, to elucidate the characteristics of cancerous interstitial tissues in RCC, we investigated the impact of molecular target drugs on these tissues. Furthermore, the effects of combination therapy with molecular target drugs with respect to inhibiting both cancer cells and cancerous interstitial tissues were analyzed as well. Finally, interstitial responses and cell differentiation need to be examined in the normal organ environment 32. Thus, in this study, orthotopic transplantation models histologically and pathologically resembling human renal cell carcinoma were also developed and evaluated.

Here, we measured the luminescence of luciferase in cancer cells using an in vivo imaging system 33 to determine the number of cancer cells. Consistent with the results based on the Ki67 LI, the direct antitumor effect of everolimus, an mTOR inhibitor, was found to be superior to that of sunitinib. However, examination of the tumor tissues excised from mice indicated that sunitinib exerted a tumor reduction effect superior to that of everolimus. Because it had been considered that everolimus primarily inhibited cancer cells, whereas sunitinib preferentially inhibited the stromal reaction, these apparently conflicting results suggested that it is necessary to analyze histological changes in tumor tissues as well as direct tumor effects following the administration of molecular target drugs.

The induction of pericytes is essential for the development of normally functioning capillary vessels 32, 34. However, prior studies have shown the intratumoral microvessels of human colorectal cancer to be covered by numerous pericytes, which protect vascular endothelial cells from antiangiogenic therapy 35, 36. Although a therapy targeting VEGF receptors on immature neovessels in tumors has attracted attention as a new approach for antiangiogenic therapy 37, 38, its therapeutic effect is insufficient because of abnormal induction of pericytes in tumor tissue. In RCC, the overall response rate to sunitinib has been reported to be greater in the high‐pericyte‐coverage group than in the low‐pericyte‐coverage group 39. Additionally, increased extracellular matrices in solid tumors elevate interstitial pressure, in turn reducing the tissue distributions of antitumor drugs 40 which suggests the importance of the role of the PDGF‐receptor signaling pathway in controlling the interstitial pressure in tumors 40, 41. This study revealed that the abnormal induction of pericytes could not be inhibited by everolimus but could be mediated by sunitinib through the inhibition of PDGF‐R*β*. Furthermore, type‐1 collagen, an extracellular matrix, was significantly reduced by sunitinib to a greater degree than by everolimus. These observations demonstrate that the use of drugs to inhibit PDGF‐receptor signaling, such as sunitinib, reduced interstitial pressure and therefore may increase the tissue distribution of concurrently administered drugs.

Finally, CAFs promote the growth and metastasis of tumors 12, 13, 14. In RCC, the interaction of CAFs with RCC cell lines stimulates tumor cell proliferation and migration 42. Our study revealed that CAFs in RCC expressed PDGF‐R*β* and were significantly inactivated by sunitinib. Similarly, the development of new blood vessels in tumors was also significantly inhibited by sunitinib.

In conclusion, everolimus significantly inhibited the proliferation of cancer cells to a greater degree than sunitinib, whereas sunitinib inhibited interstitial responses and also reduced interstitial pressure. In RCC, cancerous interstitial tissues are activated by PDGF‐BB produced by cancer cells. Our results suggest that a therapy combining everolimus and sunitinib would likely be effective toward inhibiting a cancer microenvironment consisting of both cancer cells and stromal cells. Overall, our observations indicate that a combination therapeutic strategy employing both mTOR inhibitors and tyrosine kinase inhibitors would provide a novel approach to treating RCC.

## Conflict of Interest

None declared.
